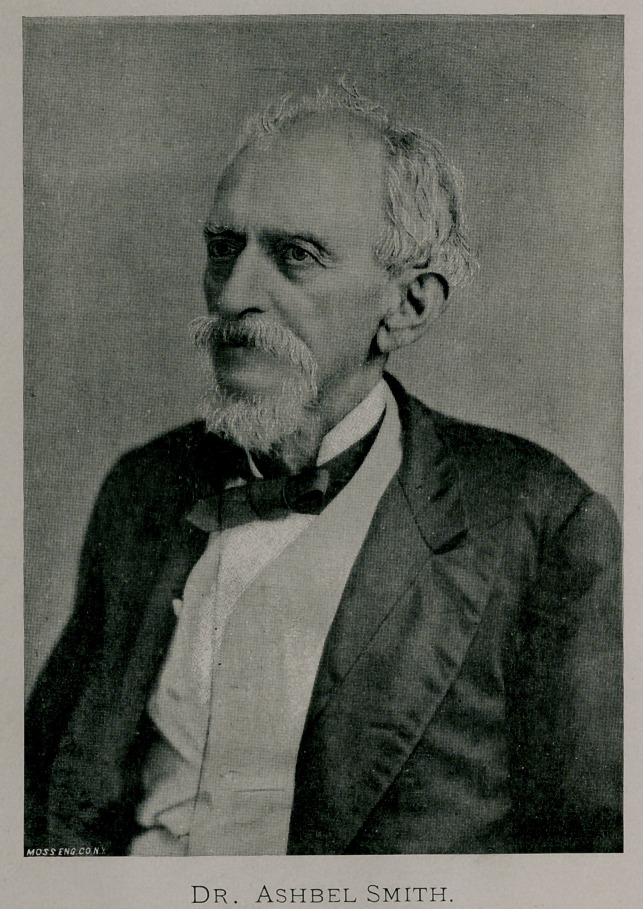# Life and Character of Dr. Ashbel Smith

**Published:** 1886-04

**Authors:** James D. Lynch


DR. ASHBEL SMITH.

DANIEL'S

### Medical ★ Journal,

PUBLISHED MONTHLY AT

AUSTIN, TEXAS.

Vol. 1.] APRIL, 1886. [No. 10.

*"Scribimus indocti, doctique!"*

### Original Articles.

☞ CONTRIBUTED EXCLUSIVELY TO THIS JOURNAL. ☜

*The Articles in this Department are accepted, and published with the understanding that we are not responsible for, nor do we indorse the views and opinions of the writers, by so doing.*

"And the good through all the ages,
Lingering in historic pages,
Ever gleam and grow immortal." * *

### LIFE AND CHARACTER OF DR. ASHBEL SMITH.

*By James D. Lynch.*

For Daniel's Texas Medical Journal.

Among all the varied compositions of human character, there is no blending of virtues so rare and so admirable as that which constitutes the patriot and philanthropist--which eliminates all idea of self from human actions, and devotes an individual to the service of his country and the welfare of his fellow-man. The memory of such men should not be permitted to perish. They belong to two worlds—they belong to Heaven for rewards, and to Earth for examples. The beacons of virtue which they have kindled and left unextinguished—the sparks which continue to flash along the pathway of departed worth, belong to every generation that afterwards crosses the shores of time. It is the sacred dnty of the people, and of the State, to cherish and preserve the memories and deeds of their eminent dead; for the achievements and characters of its great men constitute the history and the glory of a nation. Hence, to review and record the deeds and qualities of those who have enjoyed the highest esteem of their fellow-citizens, is a custom which has found sanction in every age, and to which we owe the great lessons of virtue and the beacons of individual greatness, which have shed their hallowed light upon the pathway of succeeding generations, and pointed man to a higher and nobler sphere.

If this cherished remembrance be praiseworthy—nay, glorious—in regard to those who gave honor to the hoary-headed nations of antiquity, how much more so in regard to those who, like the subject of this sketch, have rocked the cradle of their country, taught it to walk and to stand up, and took a prominent part in the establishment of a great empire.

Ashbel Smith, a Texas patriot, scientist and philanthropist, was born in Hartford, Connecticut, on the 13th of August, 1806. His family was connected with the Seymours, of Connecticut, and the Adamses, of Massachusetts, two of the oldest families in New England. His early advantages were excellent. He was placed, at an early age, in the Grammar School of his native city, and, after thorough preparation, was sent to Yale College, from which he was graduated in 1824. Being of an ardent and enterprising disposition, he entered at once upon the task of carving his own fortune, and soon after leaving college immigrated to North Carolina, and located in the town of Salisbury, where he began the study of law; but finding, at the expiration of a year, that his health was broken by the arduous application which he had bestowed upon the exacting preparation at that time required for admission to the bar, he abandoned the law, and determined to devote himself to the more congenial practice of medicine. Having attended medical lectures in New Haven, and also in the city of New York, he returned to Yale College, and graduated in the medical department of that institution. In order to acquire a yet more comprehensive knowledge of surgery and the treatment of diseases, he soon afterwards repaired to Paris, France; and having enlarged his skill and experience by his attendance on the hospitals of that city, he returned to North Carolina and began the practice of medicine in Salisbury, where his personal and professional accomplishments ordained him every success. But the star of the Young Republic of the West had taken its place solitary, but bright, in the firmament of nations;—he was attracted by its twinkling, and sympathized with the noble spirits who were still gallantly defending its shrine, and determined to go to their assistance. In June, 1837, he arrived in Texas, and was soon afterwards appointed Surgeon-General of the Texan army. In the spring of the ensuing year, he was appointed, in company with Mr. Irvin, then Secretary of State, to effect a treaty with the Comanche Indians, whose chiefs, with a hundred warriors, had visited Houston, at that time the Capital of the Republic, upon a spying or reconnoitering expedition. This they accomplished so effectually as to preserve, for a long time, the safety of the border settlements.

In December, 1838, as there was apparently no further need of active military operations, Dr. Smith resigned his position as Surgeon-General, and settled in Galveston, where he resumed the practice of his profession. In September, 1839, yellow fever appeared for the first time as an epidemic in that city. None of the physicians there had ever seen a case of the disease, and Dr. Smith found himself battling with an unknown enemy, and one whose onset it seemed almost impossible to successfully encounter; but he consecrated all his energy and skill to his efforts to stay the ravages of the fatal malady. He made strenuous endeavors to master a knowledge of its character. He made numerous autopsies, carefully observed the effects of the various remedies employed, and subsequently published a treatise, presenting the results of his investigations as to the true character and proper treatment of the disease, which was reprinted in the leading medical journals of both America and Europe, and was considered of great value to medical science.

His general practice was no less elevating and beneficent. His scientific knowledge, minute observation, and penetrating judgment enabled him to detect every climatic modification or aggravation of disease incident to the peculiar atmospheric agencies of the Gulf coast and local influences, and to discern every novel relation of cause and effect which they presented. In every subsequent epidemic he came forth, volunteered his services, and threw himself in the breach between the people and the pestilence.

Subsidiary to his professional skill, he was peculiarly fitted by nature for the functions and sphere of a physician. His high character for candor and truth, his well known philanthropy, as well as knowledge--his flowing sympathy and kind manners, reassured his patients, gained their confidence, and continually inspired them with the strength of hope--a treatment more effectual than all the pills and pellets of the laboratory, and it was the sessame of his great success.

He loved the profession of medicine, for its scientific scope, and because he saw in it only the continual realization of active benevolence. To alleviate the sufferings of humanity, to give ease to the agonized, and strength to the weak—to gladden, as well as prolong, human life, was, to his generous heart, the source of the greatest satisfaction and happiness, and he gave to it the highest exertion of his thought and action. He never sank the man in the physician; but at all times identified his feelings with the wishes, hopes, comforts, anxieties and pains of the afflicted. But he was not permitted to pursue an uninterrupted professional career. His healing skill was needed in another sphere. His country demanded his services, and his patriotism promptly responded to the call.

In the beginning of the year 1842, the foreign relations of the Texas Republic were in such a strained and unsettled condition as to seriously threaten the permanency of its independence. The United States government had positively and categorically rejected the overtures of the Republic for annexation to the Union, and Mexico was making preparation for the most formidable effort to reconquer the country; and, although France, England, Belgium and Holland had recognized the independence of Texas two years before, they now occupied an indifferent, if not an alienated, attitude. Ratified copies of the treaty with England had not yet been exchanged, and France was thrown into almost hostile relations by the action of her

*Charge d'Affairs* in Texas, M. DeSaligny, which sprang from a most trivial circumstance. A servant of the French Minister killed a pig that was depredating upon his horse trough, and the owner of the pig, who was also the proprietor of the hotel, chastised the servant; for which M. DeSaligny caused him to be arrested. He was released upon bond; and he then ordered Saligny to leave his premises. The Minister caused him to be again arrested, and he was released as before. M. DeSaligny was exasperated at what he considered inadequate punishment for the violation of his privileges, and, being unable to obtain any further satisfaction from the government, he promptly demanded his passport, and returned to France.

In this posture of affairs, Dr. Smith was appointed by President Houston, in February, 1842, Minister to the courts of France and Great Britain. This mission had for its special objects the settlement of the French embroglio, mediation between Texas and Mexico, the exchange of ratified copies of the treaty with England, and the establishment of the confidence of the European powers in the ability of Texas to maintain its independence. For these purposes President Houston, who was an excellent judge of persons, could not have made a more judicious selection than in choosing Dr. Smith. His dignified and courteous manners, his polite, emphatic and classical address, the winning suasion of his conversation, abetted by his republican simplicity, gained him a cordial and welcome consideration in every European circle, while his ardent patriotism inspired the intensity and perseverance of his efforts.

On his arrival in London, in May, 1842, he found that a strong pressure was being brought to bear upon the ministry, adverse to the exchange of ratifications of the treaties, by the anti-slavery party, which preferred the re-conquest of Texas by Mexico to the maintenance of its independence while tolerating the institution of slavery; but he promptly and courageously protested against any interferance on the part of the government of any foreign power in the domestic affairs of the Republic, and received satisfactory assurances of the absence of any such intention or desire on the part of Great Britain, and the ratified treaties were exchanged. He also found that two powerful ships of war, the Guadaloupe and the Montezuma, were being built in England for the Mexican government; that they were to carry the heaviest armament of the period, to be manned by British sailors, and commanded by two distinguished officers of the British navy, who had already obtained lief of absence for that purpose. These vessels were to be sent to Vera Cruz as soon as completed, to act in concert with a Mexican army of invasion, and lay waste the Texas coast. Dr. Smith promptly presented to the Earl of Aberdeen, the British Minister of Foreign Affairs, a strenuous and energetic protest against the injustice of permitting these vessels to leave English waters for such destination and purpose; and so earnest and cogent were his exertions that restrictions were laid on the commanders of the vessels, and they were finally sent to Mexico without men or armament; which greatly disconcerted the Mexican plans. Dr. Smith also learned while in London that the Spanish government had dispatched a man-of-war to the West Indies with instructions to be in readiness to aid Mexico, and had also promised it additional naval support. He immediately sought an interview with the Spanish Minister in London. This dignitary, however, disclaimed any knowledge on the subject, declared that he did not desire to have any, and referred him to the Captain-General of Cuba. But Dr. Smith left him with a thorough impression on his part of the correctness of his information, and the perfect knowledge of the Minister in regard to the matter.

Having accomplished the objects of his mission to London, Dr. Smith proceeded to Paris, and was received by the King and Royal Family, as he expressed it, "with quite obliging inquiries." He had carried a propitiatory letter from the Texas Secretary of State to the French court, in regard to the imagined affront to M. DeSaligny, expressing the wish that he should return. He was accordingly instructed by the French court to resume his post in Texas, and that ended the difficulty.

These events changed the whole aspect of Texas affairs, both at home and abroad. France and Great Britain now took an active and earnest interest in the permanency and welfare of the young Republic, and were willing to adopt vigorous measures to mediate peace between Texas and Mexico. But they still feared its annexation to the Union, and on the return of

Dr. Smith to London, in June, 1844, the Earl of Aberdeen said to him that they had repeatedly pressed on Mexico their good offices in favor of peace, based upon the independence of Texas; but he added: "You cannot expect us to beat the bush for the United States to catch the bird." He had previously told Dr. Smith that Great Britain desired to find in Texas a market for her merchandise "without having to climb over the United States tariff."

Prompted by these views and fears, Lord Aberdeen proposed to Dr. Smith "to pass a diplomatic act," in which Great Britain, France, the United States, Texas and Mexico should be invited to participate; but not, however, with any expectation that the United States would accept the invitation. The object of the proposed act was to establish peace between Texas and Mexico, and the permanent separate independence of Texas, of which the signatory parties were to be the guarantors.

The French government promptly accepted the proposition, the King giving his consent to Dr. Smith in person while the Texas Minister was paying him a visit of courtesy on the eve of his departure for London; and President Houston instructed his Secretary of State, Dr. Anson Jones, to forward instructions to the Texas Minister to conclude the act on the terms proposed by Lord Aberdeen. But Dr. Jones was then the President elect of the Republic, and, instead of sending the instructions, he sent Dr. Smith leave of absence to return to Texas. His failure to forward the instructions can only be accounted for upon the grounds that he desired to make the diplomatic act a measure of his own incoming administration. Dr. Smith was really opposed to the act, and advised adversely in regard to it. He feared the powerful and permanent influence to which it would subject the Republic. He likewise opposed annexation, until he saw that its consummation was a certainty. He then voted in its favor, for the reason, as he said, "that in an irreversable act he would not, in sentiment, be separated from his people." But the proposed diplomatic act was attended with potent results. It was this that caused the people and government of the United States to so suddenly and earnestly favor the policy of annexation; and it changed the whole destiny of

Texas. Had this act been consummated, Texas would to-day be an independent power; since it would have precluded her from ever surrendering any portion of her sovereignty.

The services of Dr. Smith in Europe were of great benefit to his country. His course was wise, cautious, and judicious, and the unswerving fidelity, the untiring zeal and the consummate skill with which he pursued the promotion and subserved the interests of his country, are worthy of the highest meed of praise, and places him among the most accomplished and successful diplomatists.

He was himself fully conscious of his diplomatic merits, and could not withhold a pardonable expression of his own just satisfaction. In November, 1844, when he saw that his work was accomplished and his mission finished, he requested to be recalled, and wrote from Paris to Dr. Jones, the Secretary of State: "As I am about to return home, allow me to indulge in a feeling of pride, so far as to say, that I leave behind me, at the courts to which I have been accredited, a reputation for capacity and conduct of which I am not ashamed."

In February, 1845, he was appointed Secretary of State by President Jones, and soon afterwards signed a preliminary treaty of peace effecting the recognition and independence of Texas on the part of Mexico, which had been induced by the influence of the British and French governments. This treaty was carried to the City of Mexico by Captain Sir Charles Elliot, British Minister to Texas, and was immediately approved by the President of Mexico, and ratified by a large majority of the Mexican Congress. But this peace, so long desired, came too late to affect the current of affairs in Texas. The Congress of the United States had already passed a joint resolution authorizing and soliciting annexation, and the people were too enthusiastic at the prospects of Texas becoming a State of the Union to care anything about their relations with Mexico, and Dr. Smith was burnt in effigy at Galveston for expressing his judgment in opposition to the measure.

Early in April, after the action of the United States Congress had rendered annexation a certainty, Dr. Smith was sent by President Jones to Europe, for the purpose of explaining to the governments which had interested themselves in the welfare of

Texas, the new position it had assumed in regard to annexation, and to express its gratitude by tendering to them the courtesy of a formal farewell. Yet this honorable mission was denounced by impatient annexationists as an effort to invoke the armed intervention of France and England, and even to receive the reward of treachery from them.

When these events had run their course, and annexation was consummated, Dr. Smith retired to his plantation, near Galveston, and devoted himself to the pursuits of agriculture and the pleasures of a refined literary and scientific taste. But he was not permitted to remain long in this repose; the Mexican war engaged his interest, and he accompanied the army of General Taylor throughout its campaign in Mexico. After his return, he several times represented Harris county in the Legislature. His services in this capacity were highly efficient, and made a lasting impression upon the policy and progress of the State. In 1855 he was chairman of the House committee on public debt, and always prided himself on having procured the passage of the act by which the revolutionary debt of Texas was honorably discharged. In a speech in behaif of this bill, and in opposition to the policy of scaling the debt, he said:

"Texas, Mr. Speaker, has achieved the highest military renown. From the storming of Bexar down to the brilliant gallantry of Captain Walker and his comrades, who, with sword in hand, cut their way forth and back through the Mexican army, there is nothing in the history of all the nations of the world more glorious than the renown of Texans for valor on the battle field. On that monument which stands in the porch of this capitol, which greets us every time we enter this hall, are inscribed the names of some of the heroes and of some of the battlefields of the olden time in Texas. For my own part, Mr. Speaker, I have not approved of the taste which has embraced in the inscriptions on our monuments the names of the heroes and battlefields of ancient Greece. The valor, the military renown of Texas, needs no comparisons with other nations to render them illustrious. They can stand alone; and they do stand in proud pre-eminence by the side of any that the long long line of history, ancient or modern, can furnish the world.

But, Mr. Speaker, we have another battle still to fight; another victory, I trust, is now to be achieved. It is that we place the reputation of Texas for honesty and integrity on as lofty a pedestal, and make it as glorious and renowned throughout the world, as pure, as white, as bright as her escutcheon of military valor.''

In addition to his legislative services, he filled, during this period, several positions of honor and importance which his reputation invoked. In 1852, and again in 1855, he was appointed chairman of the board of Commissioners appointed by the President of the United States to visit West Point and report the condition of the Military Academy.

When the civil war began in 1861, Dr. Smith was one of the first to volunteer his services in the field, and was elected captain of a company known as the Bayland Guards, which was attached to the second regiment of Texas infantry. He became colonel of this regiment upon the death of the gallant Rodgers, at Corinth. He was in nearly all the battles fought in Mississippi, and was wounded at Shiloh. He was in the siege and surrender of Vicksburg, and, on being exchanged, was ordered to organize a regiment in Texas, which grew into a brigade. At the time of the surrender, he commanded the defense of Galveston, and was sent by the Governor of Texas, in company with Judge W. P. Ballinger, to New Orleans, to arrange terms of capitulation with the Federal commander, and on his return made a formal surrender of Galveston to Admiral Thacher and Comomdore Sands.

He now once more returned to his plantation, which was situated almost inaccessibly at the head of Galveston Bay. Here, like the placid waters which spread their calm surface before him, as if they, too, had sought repose here after having been long lashed and billowed upon the raging bosom of the Gulf, he found that rest from the strife and turmoil of war which his advancing years now so much needed. But he said not to his country, like Simon of Athens, ''Come not to me again.'' He was still ready to respond to any call which Texas might make upon his services. In 1866, he was again elected a member of the Legislature. In 1876, he was appointed, by Gov. Throckmorton, one of the judges of the Centennial Exposition, and, in 1878, he was appointed, by President Hays, Commissioner for Texas to the Paris Exposition, and was chosen as one of its presiding officers. He was also, in 1882, President of the Texas State Medical Association, to which he was warmly attached. In 1879, he was again, and for the last time, elected to the Legislature. At the expiration of his term, he was appointed one of the Regents of the University of Texas, and held that position at the time of his death, which occurred on the 21st of January, 1886.

In attempting to analyze the character of Dr. Smith, one will be met at the threshold by a uniformity and consistency so woofed and cemented as to baffle the keenest edge of metaphysical penetration. It was composed of a blent bulk of mingling qualities and well balanced influences, guided by a ripe judgment, and expanded and rounded by the breath and spirit of culture and development; a well defined arching and blending of virtues that constitute the patriot, the philanthropist and the accomplished gentleman; qualities which can be best weighed, measured and defined when radiated into the motives and springs of action.

Dr. Smith always cherished an ardent and unselfish public spirit. He loved his country and his fellow man. As patriotic as Regulus, he would at any time have given his life for the good of his people. As faithful as Cincinnatus or Washington, he would never have accepted his own aggrandisement at the expense of the welfare of his country, and as brave as Jackson, he would never have yielded a principle without manly defense. He identified himself with every scheme of patriotism and benevolence within his reach, and he always held his purse in the open hand of charity.

His literary and scientific attainments were of a high order; indeed he was the most polished and classical scholar the writer has met with in the annals of Texas, and it would be well for some of those who claim superiority in this respect to study the weight of his ideas and his manner of expressing them. He was familiar with the varied schools of philosophy. He had listened to the voice of the ages, and studied the handwritings of the time. He had searched the museums and mausoleums of the old world, and his investigations were in the interest of truth. His religious convictions were firm and decided, and he took issue with every theory or supposed development of science, that disputed the truths of scriptural revelation. In an excellent address on the permanent identity of the human species, which, as the substitute for Mr. Webster, he delivered before one of the societies of Yale College in 1848, he drew a fine comparison between what he conceived to be the truth and fictions of science—or, rather, the truth and falsity of its interpretation. He contended that whatever may be the dogmas of casuists, or the infatuations of scientific skeptics, man is still the man of the Bible, no older, no younger, nor different. That he is the same to-day that he was in the morning of his creation, and his image is as unchangeable as the model after which he was made. The mummies of ancient Egypt, the sculptured marbles of ancient Greece and Rome, proclaim that men and women four thousand years ago, were of the same stature that they are at the present time, and that the same manly beauty and female loveliness, the same strength and grace, the same models of perfection of the human form which delighted the ancient Greeks command our admiration to-day. Man has never been known to reach the heighth of nine feet. The tallest Patagonian measured by the Spanish navigators was but seven feet one and a quarter inches in height— not as tall as a man named Porter then living in Kentucky. That the hero described by Homer as heaving a stone on the plains of Troy, which ten men could not move, in his degenerate days, was but a practical myth, and soldiers of Troy were no larger, no stronger, no braver than those of Buena Vista and the Valley of Mexico.

Nor has the race of man improved or deteriorated, either in mental powers, morals, or emotions. The countenance of the statute of Niobe expresses the same horror, mingled with all a mother's frenzied anxiety for her children that would characterize her to-day under similar circumstances, and the other members of the group portray the same similitude. That if a comparison be instituted between the mental powers of the human race in all former periods and the present time, the same equality will be discerned. Homer and Shakespeare, Euclid and Newton, Socrates and John Locke, Pythagoras and

Kepler, all exhibit in their several spheres of thought the same intellectual capacity.

In rare conjunction with the candor, depth and dignity of his discourse, Dr. Smith possessed a pulsating soul of humor and a piercing wit, which were continually excited by his good nature. But they were always wreathed with flowers and in no wise impaired the weight and influence of his views, nor the force and gravity of his expression.

He was once summoned to testify as an expert as to the cause of death on the trial of a person charged with murder. There were many ludicrous circumstances connected with the case, and in giving his testimony he said that there were several hypothesis in regard to the matter which he would present in numerical order. "For instance," said he, "there is hypothesis number one," which he proceeded to explain, "and next there is hypothesis number two," which he likewise humorously explained, and so on until he had presented and defined a half dozen or more hypotheses. The jury, in the face of positive evidence as to the killing, brought in a verdict of not guilty, and when asked how could they render such a verdict under the circumstances, the foreman replied that after hearing the evidence of Dr. Smith, the jury felt satisfied that the man died with *hypotheosis.*

Dr. Smith was exceedingly polite in his manner, precise and polished in his language, and in the company of ladies was one of the most gallant of men. He possessed a high wrought sense of honor, and being as chivalrous as Bayard, he never faltered in front of duty, he never drew back in face of responsibility, and was quick to resent any insult or indignity that outraged his sensabilities. He cared nothing for the displays and gewgaws of fashion and outward show, and his attire, while always neat, was always plain.

While attending the Congress of Physicians held in Philadelphia a few years since, he was riding on a street car, in company with Dr. Stuart, of Houston, and other gentlemen, who were dressed in the tip of style and fashion. The car was crowded and they were standing on the platform, engaged in conversation, when a lady approached without attracting their attention. The car driver, observing that Dr. Smith had a more homely appearance than his companions, seized him roughly by the arm, and cried out, "Make way for a lady!" Upon which Dr. Smith jerked a small knife from his pocket and pointing it ominously at the throat of the astonished driver said to him, in the suppressed, earnest tones of wounded honor, "Dare you, sir, to teach me how to be polite to a lady!"

The domestic habits of Dr. Smith were frugal and abstemious, and it was this temperate regimen that enabled him, though of slender physique, to preserve his health and vigor to the age of four score years. He was always contented and all his surroundings were lighted up by the sunshine of his intellect and cheerful temper. He was never married; yet he possessed not the peculiar eccentricities which usually belong to those who grow old in celibacy. If his sentiments were never soothed and softened by the touch of conjugal affection, they bore the silent chastening of a sad memory, wrought by the living image of a first and only loved, and never forgotten one. After his death a small package was found in careful preservation, upon which were inscribed the words, "Never to be opened, but to be placed over my heart after death." The solemn injunction was sacredly observed, and the little package sleeps with him in his bosom. It was said to contain the picture of a young lady he loved in his youth in Connecticut, and a letter written with her blood; but the secret lies buried with him, and there it will remain until the reminescenses of earthly things are called up in the reunion of eternity.

Dr. Smith was an earnest friend to the cause of public education, and was a vigorous advocate of normal schools and the interest of the State University. He appreciated the fact that the virtue and intelligence of the people form the pillars of republican government, and that a people to be free must have a knowledge of their rights and the duties they owe to society.

His views on all subjects were carefully formed from study and reflection; hence he was firm in his convictions, and as to a question of duty he was utterly immovable. He was one of those *justi ac tenaces propositi viri* of Horace, whom neither the thunders of Jove, the storms of Boreas, the frowns of Rulers, nor the applause of the populace can shake from the fixed purpose of their firm resolve; and this solidity of mind and immutible determination he exercised in all the relations of life. A few years previous to his death, he was bitten by a rattlesnake, and he conceived that iodine was the proper remedy for the poison. He could not be induced to seek any other means of relief, and persevered in its use until he cured himself with that drug alone.

He had no fears of death. He viewed it as simply a change from old to new, from age to youth, from darkness to light, from eve to morn, for those who walked in the path of faith, a transition from this to a better world; and he died as he had lived, like a Christian and a philosopher.

His remains were reverentially escorted from his former home on Galveston bay to Austin, where they were placed in state, and every honor and respect was paid to his obsequies. They were then deposited in the State cemetery by the side of many of his old friends and compatriots, who had been laid there before him.

## Figures and Tables

**Figure f1:**